# Entrance and origin of the extracranial vertebral artery found on computed tomography angiography

**DOI:** 10.1038/s41598-022-19497-7

**Published:** 2022-09-10

**Authors:** Xueting Yi, Ping Xie, Lianwei Zhang, Fengxia Lu, Hao Chen, Kefu Liu

**Affiliations:** Department of Medical Imaging, Affiliated Suzhou Hospital of Nanjing Medical University, Gusu School of Nanjing Medical University, No.242, GuangJi Road, Suzhou, 215008 Jiangsu China

**Keywords:** Anatomy, Neurology

## Abstract

To investigated morphological variability of vertebral artery (VA) origin and its entrance level into cervical transverse foramina by computed tomography angiography (CTA). To retrospectively investigated CTA of 223 subjects (446 VA courses). Investigated were origin of the VA and its level of entrance into vertebral transverse foramen with notification of the sex and side of variation. The VA entered the C6 transverse process in 91.70% of specimens (409 out of 446 VA courses). Abnormal entrance of VA was observed in 8.30% of specimens (37 VA courses), with the level of entrance into the C3, C4, C5, or C7 transverse foramen at 0.22%, 2.47%, 4.71% and 0.90% respectively. Comparably, the overall variability of abnormal origin of VA was 1.57% (7 out of 466 VA courses), in which the left vertebral arteries all arose from aortic arch. The variation rate of vertebral entrance rose up to 50% in abnormal origin subgroup. When comparing subgroups of subjects with normal and abnormal origin, there was significance difference in the frequency of entrance variation in the level of transverse foramen (*p* < 0.001). Abnormal entrance and origin of VA were observed in 8.30% and 1.57% of VA courses, which can be accurately appeared by CTA. Regarding to the subgroups of abnormal origin, the frequency of entrance variation was significantly increased in the level of transverse foramen compared to that of normal origin.

## Introduction

The anatomical variation of vertebral artery (VA) is essential to clinics and anatomy. Accurate understanding in the variations of vertebral artery are regarded as necessary with the development and popularization of endovascular interventions, anterior decompression of the cervical spine, neck dissection and stellate ganglion block.

The anatomical variation of VA, including its origin and its level of entrance into transverse foramen, has been widely investigated, and even some general information has been published in standard atlas, textbooks and the Internet^1^. However, the recent significant findings indicated that the differences of VA variations among different regions raised the issue of potential regional, ethnic and even environmental factors^2^. Up to now, there is a lack of original data available for regions of China.

Therefore, our current study investigated morphological variability of VA origin and its entrance level into cervical transverse foramina in population of Suzhou in China.

## Materials and methods

This retrospective study has been approved by the Institutional Review Board of Suzhou Municipal Hospital, Nanjing Medical University, Suzhou, China. The requirement for informed consent was waived owing to the retrospective nature of the study. The study was conducted in accordance with the Declaration of Helsinki, and the protocol was approved by the Ethics Committee of Suzhou Municipal Hospital.

The study was conducted as a retrospective single-center study at the Affiliated Suzhou Hospital of Nanjing Medical University. From January 2019 to August 2019, 300 patients who resided in Suzhou of China and were suspected of having cerebrovascular diseases were enrolled in this study. We excluded 67 patients with current or previous diagnosis of cerebrovascular accidents, cervical injuries, stenotic atherosclerotic lesions (over 50% decrease of VA diameter) and severe spondylarthrosis, considering vascular stenosis leading to inadequate filling of contrast medium and cervical trauma or surgery affecting the normal anatomical structure of the transverse foramen. Additionally, the other 10 patients were excluded with artifact in computed tomography angiography (CTA). Ultimately, we investigated CTA of 223 subjects (155 men and 68 women).

All imaging-generation and visualization measurements were performed in the Clinical Radiology Department, Affiliated Suzhou hospital of Nanjing Medical University (Suzhou, China). Before being test, volunteers should meet the following requirements: (1) fasting, (2) no history of severe allergy to contrast medium, (3) normal renal/hepatic function. Subjects were placed in a supine position with their arms alongside the body, head and neck kept at a neutral position. The imaging examination was performed on a Multi-slice computed tomography scanner (Philips Brilliance iCT Holland) with the scanning protocol as follows: 120 kVp, 153 mAs, beam collimation 128 × 0.625 mm, gantry rotation time 0.5 s, section thickness of 0.9 mm, pitch 0.758 and reconstruction interval of 0.45 mm. During the procedure, infused 50 mL of nonionic iodinated contrast was followed by 30 mL saline and injected via a double power injector into the patient’s antecubital vein (5 mL/s).

Images were analyzed at a workstation (Intellispacsportal 9.0; Philips). The VA’s origin and level of entrance into vertebral transverse foramen were recorded, as well as the sex and side of variation. All the data was measured by 2 experienced radiologists with independent double-blind method, and a consensus was reached finally.

Statistical analysis was performed using statistical package for the social sciences (SPSS) version 22.0 (IBM Corporation, Somers, NY, USA) statistical software. The data were expressed as means ± standard deviation (SD). The quantitative data were examined by Kolmogorov Smirnov test for normality. Independent “t” test was performed to compare the different variables regarding side. Chi-Square test and Fisher's exact test (when one or more of the cell counts in a 2 × 2 table is less than 5) were used to determine if there was a significant relationship between different variables. p value < 0.05 was considered to be significant.

## Results

Generally, the mean age of patients was 65 ± 13 years (range from 88 to 27), and the male-to-female ratio was 2.3:1 (310 vs. 136) in the current study.

**Figure 1:**
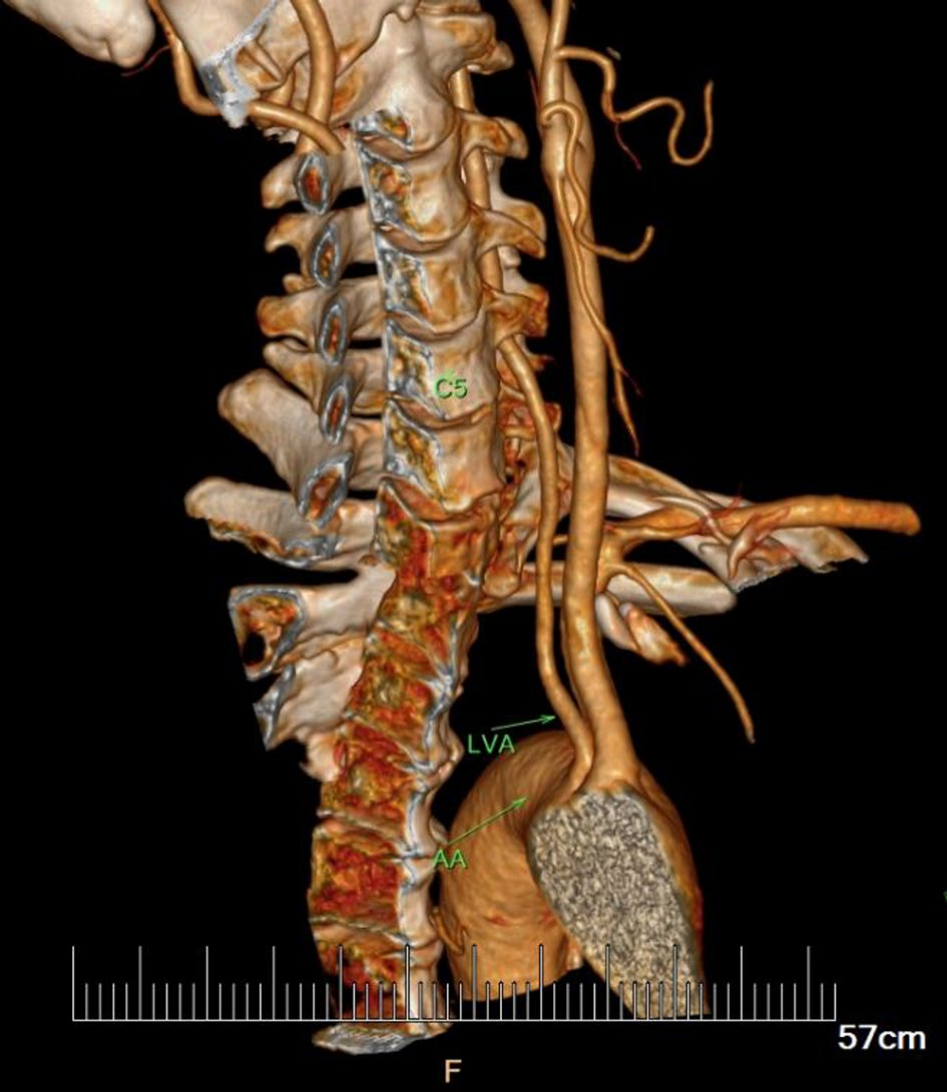
Typical case of subjects with LVA arising from aortic arch. The distal segment enters the transverse foramen at C5 level higher than normal.

**Figure 2:**
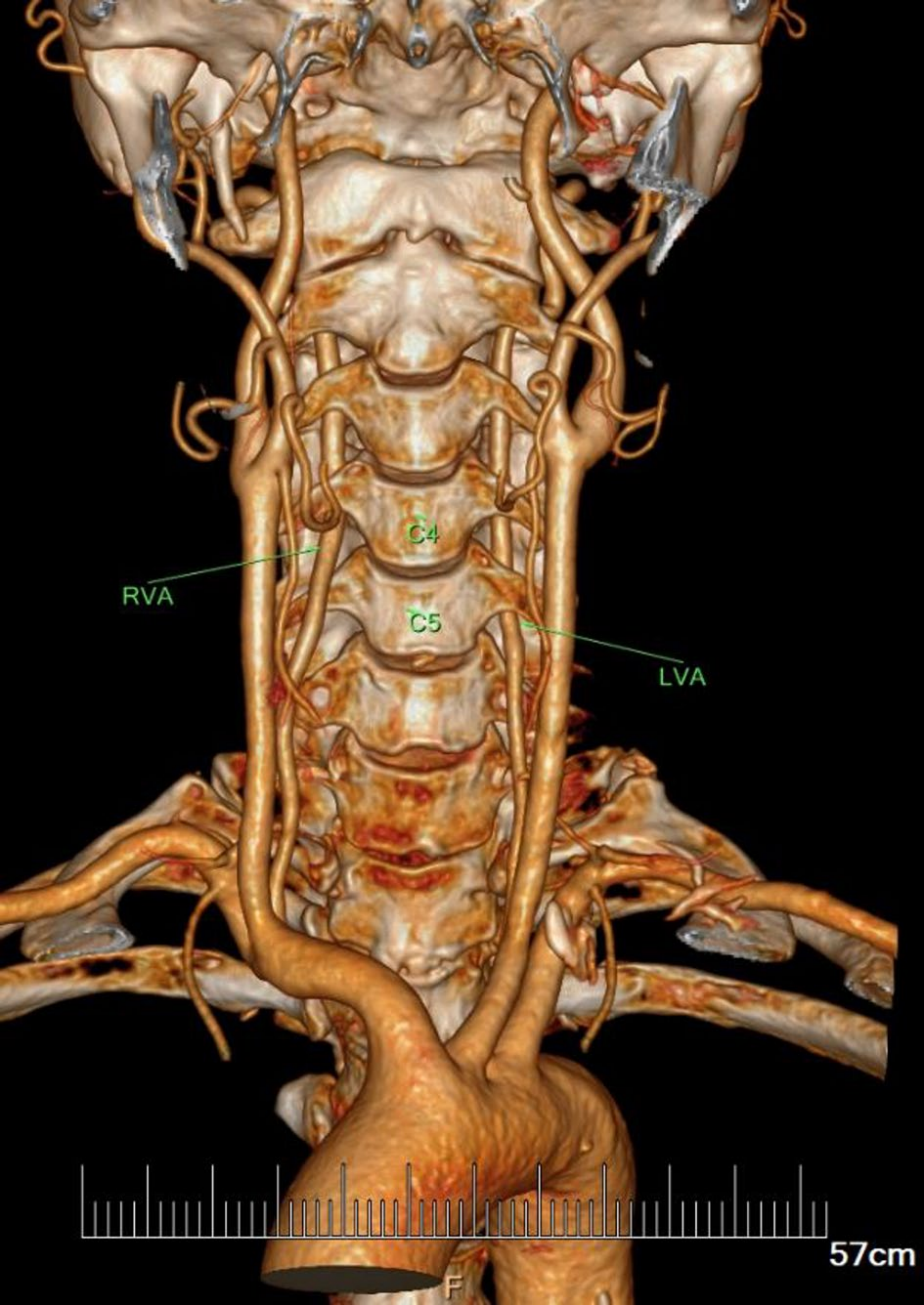
Typical case of the subjects with anomalous entrance at a higher level into the transverse foramen than normal (LVA: C5; RVA: C4).

**Figure 3:**
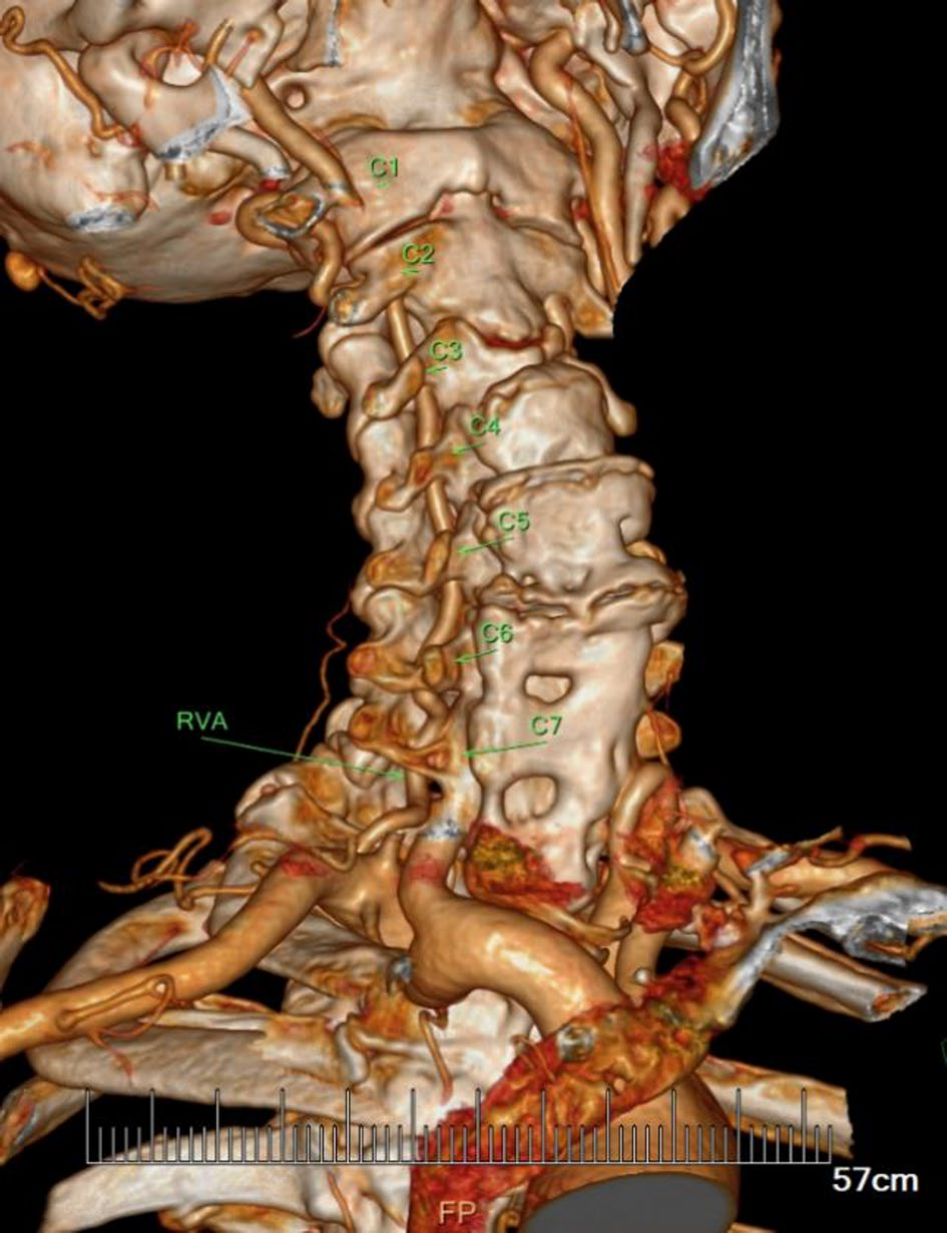
Typical case of the subjects with anomalous entrance at a lower level into the transverse foramen than normal (RVA: C7).

In the current study, the VA entered the C6 transverse process in 91.70% of specimens (409 out of 446 VA courses), in which 69.43% were males and 30.56% were females (Table 1). Abnormal entrance of VA was observed in 8.30% of specimens (37 VA courses), with the level of entrance into the C3, C4, C5, or C7 transverse foramen at 0.22%, 2.47%, 4.71% and 0.90% respectively (Table 1, Figs. 1, 2, 3).

**Table 1:** Origin and entrance into the transverse foramen of the VA in overall population (223 subjects; 446 courses).

Entrance into the transverse foramen	Total (N = 446)	Origin of VA							
		Subclavian artery				Aortic arch			
		Right		Left		Right		Left	
		Male	Female	Male	Female	Male	Female	Male	Female
C3 lever	1 (0.22%)								1
C4 lever	11 (2.47%)	7	3			1			
C5 lever	21 (4.71%)	6	2	3	5	1		4	
C6 lever	409 (91.70%)	134	62	144	62	4	1	2	
C7 lever	4 (0.90%)	2		2					

Percentage values are shown in parenthese of overall population.

There was non-significant statistical difference in the frequency of entrance variations of right vertebral artery (RVA) and left vertebral artery (LVA) (22 vs. 15, *X*^2^ = 1.444*, p* = 0.229). In male cases, there was non-significant statistical difference in the frequency of entrance variations of right VA(RVA) and left VA(LVA) (17 vs.9, *X*^2^ = 2.687, *p* = 0.101), nor in female cases (5 vs. 6, *X*^2^ = 0.099, *p* = 0.753).

There were 14 VA s originated from aortic arch. Comparably, we observed that the overall variability of abnormal origin of VA was 1.57% (7 out of 446 VA courses), which LVAs all arose from aortic arch (Fig. 1). Notably, seven subjects (6 males and 1 female) of the left VA originated from aortic arch, where the variation rate of vertebral entrance rose up to 50% in corresponding subgroup. Among these anomalous entrance in patient with abnormal origin, one entered the transverse foramen at the level C3, one at level C4, five at level C5, respectively. When comparing subgroups of subjects with normal and abnormal origin, there was significance difference in the frequency of entrance variation in the level of transverse foramen (30/432 vs. 7/14, *p* < 0.001).

## Discussion

We could recognize the location of the entrance and origin of the VAs in all our cases due to high-quality CTA, which is a noninvasive and easy imaging technique and is very important for surgery. The entrance and origin of the VA is essential to neck tumor resection as vascular damage may occur when VA goes through the abnormal region, which is sometimes neglected by the surgeon. Additionally, the entrance of the vertebral artery is of significance to the stellate ganglion block. The block is commonly performed on the anterior tubercle of C6 transverse process. When the VA enters the foramen of the C5 transverse process or higher, exposing the VA between the cervical transverse processes, the VA is vulnerable to be located in the needle path. Unawareness of such an aberrant VA may cause life-threatening events^3^. The origin of the vertebral artery is also very important in the placement of the aortic arch stent during peripheral vascular intervention, otherwise could produce injury and local occlusion of the vertebral artery^4^.

In the course of reviewing papers, we found an interesting issue that there were some differences in the rate of variation of vertebral artery inlet in various reports. The variability of anomalous entry level we found was 8.30%, compared to 12.5% in Republika Srpska^5^, 15.7% in Germany^6^, 5.75% in Isreal^7^, 7.0% of LV and 6.2% of RV in Japan^8^, 8.7% in Taiwan, R.O.C^9^, 5.3% of LV and 0.8% of RV in Thailand^10^, 5.1% in Korea^11^, 8%of LV and 6.6% of RV in another study of Korea^12^, 7.5% of LV in Turkey^13^. This could be explained by sample sizes and structure differences. However, this difference is also likely to be related to the differences in the embryonic development of the vertebral artery caused by racial differences. Our study fills in the gap in Chinese population and sheds lights on further related research with a reliable foundation.

Literature shows the frequency of origin of the left VA from aortic arch in the range of about 1–3%^14^. Our results indicated that the overall variability of origin of VA was 1.57%. In addition, we found that the origin variation of vertebral artery all occurred on the left side. Notably, our study found that when the LVA arose from aortic arch, the variability of the entrance level reached to 50%. When comparing subgroups of subjects with normal and abnormal origin, there was significance difference in the frequency of entrance variation in the level of transverse foramen, which was consistent with the Japanese finding that anomalous origin and anomalous entry level into the transverse foramen correlated strongly^8^. Similarly, Lin et al.^9^ reported that there was a significantly higher rate of anomalous entrance level (C4 or C5) to normal C6 entrance level of the patient with abnormal VA origin. Meila et al*.*^6^ claimed that LVA originating from the aortic arch would never enter the transverse foramen at the level of C6, which turned out to enter at a higher level at C4 or C5 with an aortic arch origin proximal to the subclavian artery, or at C7 with an aortic arch origin distal to the subclavian artery respectively. However, their view caused contradictory when taking our findings into consideration. Interestingly, our finding revealed that 50% of patients with anomalous LVA origin was observed the correlated anomalous entry level into the transverse foramen, while the rest 50% of them was found with normal entry level; whereas Lin et al*.*^9^ showed that 4.34% of patients with anomalous LVA origin was observed with normal entry level. The large difference in proportion between our study and theirs has aroused our interest in the investigation of embryonic development.

A brief review of embryology of the aortic arch and great vessels makes a better understanding of these variations^6^. The embryo develops 6 pairs of matched aortic arches^15^. These aortic arches undergo selective apoptosis, and the remaining branch vessels form the aortic arch and large vessels. During this process, anatomical variation can be formed occasionally. The first and second aortic arches (I and II) degenerates. The paired third arch (III) forms the first part of the bilateral internal carotid artery. The proximal right fourth arch (IV) remains to serve as the origin of the right subclavian artery to the internal mammary artery, while the distal part of the right fourth arch regresses. The left 4 arch (IV) degenerates and forms small segment of the adult arch between the origin of the left common carotid artery and the left subclavian artery. The fifth arch (V) either recedes or is not fully formed. The sixth arch (VI) forms the pulmonary arch and develops into the right pulmonary artery and artery catheter^16^.

VA is the longitudinal anastomosis of segmental meristem artery^17^. The bilateral VAs usually develop from the distal end of the seventh intersegmental dorsal arteries. The proximal two-thirds of subclavian artery to the level internal thoracic artery is formed by the left seventh dorsal intersegment, while the distal third of the proximal subclavian artery is formed by the right seventh intersegment. Vascular malformation originated from abnormal anastomosis at any time during the embryonic development of the arch. The timing and location of the anastomosis will determine the ultimate origin of the variation after grown-up. Notably, VA can be anastomosed with ascending and deep carotid arteries. The difference of the level of vertebral artery entrance is associated with the dominance of the ascending or deep cervical artery anastomosis.

In order to better understand the origin and variation of the vertebral artery, we speculated the development pattern based on the findings of Lin et al.^9^. RVA was normally generated from the first part of the subclavian artery, and flowed into the transverse foramina in the sixth cervical vertebra C6, then passed through the transverse process of C6 to the first cervical vertebra C1, and eventually extended from C1 to the intracranial segment via foramen magnum. However, anatomical variation of the LVA origin was observed. Anastomose occurred between LVA and ascending carotid artery, which causes LVA enter at the level of C5 if the ascending carotid artery took the lead, then climbed up through from C5 to C1, and eventually from C1 to the intracranial segment.

Specifically, when the longitudinal intersegmental anastomosis was dominated by ventral group such as ascending cervical artery, the anomalous VA originating from 6th cervical segmental artery ran along with the ascending cervical artery and shifted up to the entry level at C5 or above into the transverse foramen. On the contrary, when the longitudinal intersegmental anastomosis was dominated by dorsal extraspinal group such as deep cervical artery, the anomalous VA ran along with deep cervical artery and shifted down to the entry level at C6 into the transverse foramen.

## Conclusion

Abnormal entrance and origin of VA were observed in 8.30% and 1.57% of cases, which can be accurately appeared by CTA. Regarding to the subgroups of abnormal origin, the frequency of entrance variation was significantly increased in the level of transverse foramen compared to that of normal origin.

## Supplementary Information

Supplementary Information.

## Data Availability

All data generated or analysed during this study are included in this published article (and its Supplementary Information files).
